# Proteomics Studies Suggest That Nitric Oxide Donor Furoxans Inhibit In Vitro Vascular Smooth Muscle Cell Proliferation by Nitric Oxide-Independent Mechanisms †

**DOI:** 10.3390/molecules28155724

**Published:** 2023-07-28

**Authors:** Loretta Lazzarato, Laura Bianchi, Annapaola Andolfo, Agnese Granata, Matteo Lombardi, Matteo Sinelli, Barbara Rolando, Marina Carini, Alberto Corsini, Roberta Fruttero, Lorenzo Arnaboldi

**Affiliations:** 1 Department of Drug Science and Technology, Università degli Studi di Torino, Via Pietro Giuria 9, 10125 Torino, Italy; loretta.lazzarato@unito.it (L.L.); barbara.rolando@unito.it (B.R.); roberta.fruttero@unito.it (R.F.); 2 Functional Proteomics Laboratory, Department of Life Sciences, Università degli Studi di Siena, Via Aldo Moro 2, 53100 Siena, Italy; laura.bianchi@unisi.it; 3 Proteomics and Metabolomics Facility (ProMeFa), Center for Omics Sciences (COSR), IRCCS San Raffaele Scientific Institute, Via Olgettina 60, 20132 Milano, Italy; andolfo.annapaola@hsr.it; 4 Department of Pharmacological and Biomolecular Sciences “Rodolfo Paoletti”, Università degli Studi di Milano, Via Balzaretti 9, 20133 Milano, Italy; agnese.granata@unimi.it (A.G.); matteo.lombardi11@gmail.com (M.L.); matteo.sinelli@gmail.com (M.S.); alberto.corsini@unimi.it (A.C.); 5 Department of Pharmaceutical Sciences “Pietro Pratesi”, Università degli Studi di Milano, Via Mangiagalli 25, 20133 Milano, Italy; marina.carini@unimi.it

**Keywords:** atherosclerosis, smooth muscle cell proliferation, nitric oxide, furoxans, small ubiquitin-related modifier 1, proteomics

## Abstract

Physiologically, smooth muscle cells (SMC) and nitric oxide (NO) produced by endothelial cells strictly cooperate to maintain vasal homeostasis. In atherosclerosis, where this equilibrium is altered, molecules providing exogenous NO and able to inhibit SMC proliferation may represent valuable antiatherosclerotic agents. Searching for dual antiproliferative and NO-donor molecules, we found that furoxans significantly decreased SMC proliferation in vitro, albeit with different potencies. We therefore assessed whether this property is dependent on their thiol-induced ring opening. Indeed, while furazans (analogues unable to release NO) are not effective, furoxans’ inhibitory potency parallels with the electron-attractor capacity of the group in 3 of the ring, making this effect tunable. To demonstrate whether their specific block on G1-S phase could be NO-dependent, we supplemented SMCs with furoxans and inhibitors of GMP- and/or of the polyamine pathway, which regulate NO-induced SMC proliferation, but they failed in preventing the antiproliferative effect. To find the real mechanism of this property, our proteomics studies revealed that eleven cellular proteins (with SUMO1 being central) and networks involved in cell homeostasis/proliferation are modulated by furoxans, probably by interaction with adducts generated after degradation. Altogether, thanks to their dual effect and pharmacological flexibility, furoxans may be evaluated in the future as antiatherosclerotic molecules.

## 1. Introduction

Atherosclerosis is a multifactorial disorder characterized not only by lipid accumulation, but also by several other components [1,2,3,4,5]. Among these, in this work, we focus on the control of smooth muscle cell (SMC) proliferation and nitric oxide (NO).

### 1.1. SMCs and Atherosclerosis

In physiological conditions, SMCs are the main cell population of the arterial medial layer, where they maintain functional and structural integrity, thanks to the contractility and synthesis of the extracellular vascular matrix [6]. These SMCs are characterized by a contractile phenotype, fusiform shape, and high expression of genes codifying for myofilaments and structural proteins such as SMC myosin heavy chain (MYH11), 22-kDa SMC lineage-restricted protein, alpha-actin-2 (ACTA2), smoothelin; they are also differentiated and respond to vasoconstricting and vasodilating stimuli, but are silent towards mitogens [7].

According to the “response-to-retention” hypothesis, during the early stages of atherosclerosis [2,3,5,8,9] after endothelial injury and consequent NO loss, circulating low density lipoproteins (LDLs) penetrate the subendothelium and, once retained by proteoglycans, become oxidized and stimulate SMC migration and proliferation. Migrated SMCs are rather different compared with medial ones, since they are characterized by a synthetic phenotype [10], are round in shape, are enriched in Golgi and ribosomes, express low ACTA2, show enhanced genes codifying for growth factors and cytokines, are much less differentiated, proliferate at a higher rate, accumulate lipids, secrete chemokines, cytokines and growth factors, promote inflammation [11] and extracellular matrix (ECM), but also ECM-degrading enzymes as matrix metalloproteinases (MMPs) [12]. MMPs play a pivotal role, since they degrade the basal membrane matrix around SMCs, facilitating their migration to intima [13,14,15]. Migrated SMCs accumulate lipids, thus becoming foam cells and acquiring macrophage markers and properties [1]. Several in vitro and in vivo studies suggest that the Kruppel-like factor 4 (KLF4) plays an essential role in this phenotypic transition [16,17,18,19]. Animal models document that SMC-derived foam cells could account for as much as 50% of lesional foam cells [20].

In advanced stages of atherosclerosis, SMC may also adopt mesenchymal stem-, myofibroblast-, and osteochondral-like phenotypes, the latter contributing to calcium and phosphate deposition [19,20,21]. Compelling evidence demonstrated that not only SMCs produce ECM, which is also secreted by endothelial cells and macrophages [22], but they may also undergo apoptosis/necrosis, generating a “necrotic core” composed of cholesterol and cell debris that increases the likelihood of lesion rupture [22]. SMC and endothelial cell senescence may also affect lesion stability by amplifying inflammation and producing proteases against the fibrous cap [23,24]. Therefore, based on these data, the inhibition of SMC proliferation may be beneficial since it may contribute towards halting, or at least decreasing, this cascade of events [7,25].

### 1.2. Nitric Oxide (NO) and Atherosclerosis

From a physical–chemical point of view, NO is a lipophilic gas with a half-life of a few seconds, which exists in an intermediate state of oxidation, conferring its abilities both in reducing and oxidizing substrates [26].

While at low concentrations, NO displays protective and pro-survival effects, its overproduction may be both detrimental, inducing apoptosis and toxicity [27], and beneficial, since it is lethal for bacteria, parasites, and tumor cells at high concentrations due to reactive nitrogen species, which harm cell membranes, endoplasmic reticulum, mitochondria, nucleic acids, and proteins/enzymes [28].

Biologically, NO is an endogenous key regulator of cell function, since it activates/deactivates transcription factors, gene transcription, and mRNA translation [29]. The transient production of low nanomolar NO is essential for various biological processes, mainly due to the nitrosylation of the soluble guanylyl cyclase. The NO radical also reacts with different partners, such as the -SH groups of cysteine in peptides, resulting in the formation of S-nitrosothiols. Moreover, NO also directly reacts with oxygen species, producing nitrogen oxides, which result in the post-translational regulation of protein structure and function [29,30]. All of these properties translate into the NO-induced prevention of platelet–leukocyte activation/adhesion, the inhibition of vascular SMC proliferation and migration, and the modulation of inflammatory reactions. 

In mammalians, NO may be enzymatically synthesized from L-arginine by three NO synthases (neuronal, endothelial, and inducible; nNOS, eNOS, and iNOS), with the production of NO and L-citrulline or by nitrite disproportionation/reduction after tissue damage, following the reaction [29,31,32]:

3HNO_2_ ⇋ H^+^ + NO_3_^−^ + 2NO + H_2_O

nNOs and eNOS are constitutively present in neurons and endothelium, respectively, and generate NO in a controlled and transient fashion (at nM concentrations). In physiological conditions, nNOS is also expressed in vascular SMCs [33,34] and endothelium [35]. In fact, in nNOS-KO mice, which display increased neointimal formation and constrictive vascular remodeling, restoring nNOS suppresses atherosclerotic vascular lesion formation [29,36,37].

On the other hand, eNOS is present in normal aorta, adventitia, neutrophils, monocytes, and in atherosclerotic plaques (SMCs included) [38]. iNOS, primarily detected in cytokine-induced macrophages [39] after inflammation and oxidative stress, produces NO continuously at higher and potentially toxic concentrations (µM). This condition may be further induced by fluid shear stress. In normal vessels, NO synthesized by iNOS is a major regulator of vasal tone and inflammation and its reduced expression/activity is compensated by that of nNOS in human atherosclerotic lesions [40].

NO plays a fundamental antiatherogenic role since, when synthesized by intact endothelial cells, it modulates vascular tone, SMC proliferation, and oxidative processes [29,32]. Moreover, NO also possesses antioxidant properties, since it increases the activity of the enzymes able to remove O_2_^•−^ and upregulates extracellular superoxide dismutase expression, thus preventing its O_2_^•−^-mediated degradation [41,42].

On the other hand, chemical and mechanical stimuli typical of the atherosclerotic process may affect endothelial function, thus depleting NO and finally resulting in atherothrombotic phenomena. In an intact vessel, most of the NO comes from eNOS activity; even if acetylcholine, histamine, thrombin, serotonin, ADP, norepinephrine, or bradykinin stimulate NO synthesis and release, the main physiological stimuli are dynamic forces generated by blood flow on the vessel surface [43,44].

NO produced by eNOS regulates vasal tone, endothelium apoptosis, platelet function, NF-kB-mediated inhibition of leukocyte adhesion via the reduction in the genes encoding for chemokines and the adhesion molecules (MCP-1, VCAM-1), inhibits LDL oxidation, and regulates SMC proliferation (see next paragraph). NO also cGMP-dependently inhibits adhesion, migration, and platelet aggregation, while it increases endothelial cell proliferation and, at high concentrations, may cause apoptosis [31,45,46,47,48].

Atherosclerotic vessels are also characterized by increased NO inactivation via the abnormal production of oxygen radicals [8,46,49,50]. Several studies demonstrated an association between insufficient NO production or activity and atherosclerosis development and progression in humans, while studies in KO mice have shown that eNOS and nNOS are atheroprotective, with iNOS being controversial [38,51,52,53].

It is also true that NO tends to increase along with plaque progression thanks to the activation of the iNOS by platelet-activated macrophages, but the effects of this NO may be deleterious due to the production of ONOO^−^, which modifies LDL, peptides, and nucleic acids and activates signaling pathways [54,55,56].

### 1.3. NO and SMC Vasodilation and Proliferation

Physiologically, NO produced by eNOS in endothelial cells diffuses into adjacent SMCs, where it activates soluble guanylyl cyclase, Ca^2+^ channels, and protein Kinase G I, finally decreasing Ca^2+^ flux, leading to vasodilation [57,58,59,60,61].

Moreover, NO inhibits SMC proliferation through a cGMP-dependent and a cGMP-independent mechanism [31]. In the first case, NO catalyzes the formation of cGMP, which increases cAMP, determining protein kinase A activation that decreases intracellular Ca^2+^ and inhibits Raf-1, an activator of MAP kinase signaling, which regulates DNA biosynthesis [60,61,62]. The NO-cGMP signaling plays a crucial role in plaque development, since its activation promotes a shift in SMCs toward a contractile phenotype, characterized by reduced proliferation. Alternative effectors are the cGMP-dependent inhibition of c-myc and the overexpression of p21, both involved in cell-cycle control [20,32,63,64,65].

The second mechanism is dependent on the direct inhibition of arginase and ornithine decarboxylase, both enzymes involved in polyamines (spermidine and spermine) production [66], which both stimulate RNA, DNA synthesis, and SMC proliferation [31].

### 1.4. NO Donors

It is worthwhile to mention that in atherosclerosis the functionality of exogenous NO is almost intact, thus allowing the administration of NO donors to vicariate its beneficial effects. Nevertheless, despite various pharmacological means being available, several issues have dampened large-scale applications [28]. The duration of NO exposure time and kinetics are key determinants in its biological applications; moreover, its erratic solubility in water, extremely short-half life, propensity to diffusion, and its oxidation to the membrane permeant radical nitrogen dioxide (^•^NO_2_) limit its therapeutic use due to lack of pharmacological efficacy or even possible adverse effects [67,68]. To overcome this problem, several chemical strategies have been developed to release NO in a controlled manner. Designing NO donors is quite difficult both due to the “two faced” (beneficial and detrimental) nature of NO and to the relatively poor drug-like properties of NO mimetics [28,32,68]. The most striking difference between endogenous and exogenous NO is the fact that the first is a free radical, while the latter exists in three different, but related redox species, each with distinct chemical and reactive properties: free radical (NO^•^), nitrous cation (NO^+^), and nitrosyl anion (NO^−^) [69]. 

The earliest discovered NO donors include classic vasodilators and anti-angina pectoris agents such as organic nitrates (nitroglycerin, erythrityl tetranitrate, and isosorbide dinitrate), nitrites, and nitrite thiol esters. N-diazenium diolates (NONOates), thanks to their parent structure, are very interesting, since their conjugation with enzymes or metabolite response groups can regulate and control NO release. Compared with NONOates, nitrosothiols do not spontaneously release NO, but, after decomposition, may produce NO (e.g., by Cu^+^, ascorbic acid, UV light, heat, and enzymes such as superoxide dismutase) [70,71,72]. It is worthwhile to mention that, from a pharmacological point of view, nitrosothiols and NONOates are used due to their ability to quantitatively release NO radicals when dissolved in physiological solutions [28,68].

Among synthetic NO donors, furoxans (1,2,5-oxadiazole 2-oxide) are an old and well-known heterocyclic system with an intriguing chemistry. In the recent past, a renewed interest in furoxan ring derivatives arose, since under the action of thiols, they activate the soluble guanylylcyclase, thus releasing NO and acting as vasodilators [73,74,75,76]. Several furoxans have been systematically studied for their NO-dependent vasodilating properties. Their potencies are extremely modulable depending on the substituent in position 3 of the ring, and their NO-releasing ability can be abolished by the NO scavenger HbO_2_^++^. These properties have also been confirmed in animal studies, in which furoxans have been administered as antihypertensive molecules [77,78,79]. Concerning their flexibility as a pharmacological tool, the presence of electron-withdrawing groups on the ring, in particular at the 3-position, increases the rate and amount of NO production, but the exact mechanism is unknown and only speculative hypotheses have been proposed [28,80].

**Scheme 1:**
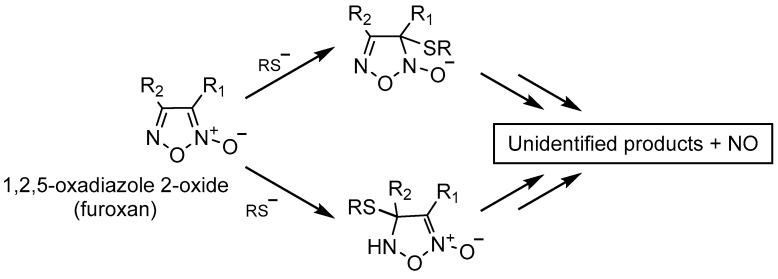
Proposed mechanism of furoxans’ NO release and of thiol bioactive adduct formation; R_1_ = group in position 3; R_2_ = group in position 4.

Likely, the first step should imply an interaction of the electrophilic 3-position of the ring with the nucleophilic–SH group (position 4 may also react with thiols), followed by ring opening and NO release. Moreover, Feelisch et al. [81] demonstrated that furoxans, when incubated in saline plus thiols, not only release NO, but also produced dioxime derivatives, nitrites, nitrates, and S-nitrosothiols. Reversible or irreversible thiol adducts may also be produced, thus contributing to furoxans’ pharmacological and toxicological properties [82], as shown in the following scheme (Scheme 1).

Finally, a number of hybrid products have been obtained by combining, via appropriate spacers, the 3-phenylsulfonylfuroxan moiety with that of anti-tumor derivatives, and these compounds have been tested for their antiproliferative activity in sensitive cancer cell lines [82].

Based on these premises:

(a) We ran preliminary screening on the possible antiproliferative effects exerted by different NO donors and, among these furoxans, on human and rat vascular SMCs. After determining that furoxans are really effective, to understand the mechanism of action of this effect, we tested whether their ring-opening abilities and the nature of the substituent in position 3 may affect these properties. To address this point, we also tested furazans (compounds structurally and chemically related to furoxans devoid of NO-releasing properties).
(b) We also aimed at comprehending whether these molecules may possess a phase-specific effect on the cell cycle, since it is known that NO causes a block in the G1-S phase [83]. For this purpose, we utilized the incorporation assay of radiolabeled thymidine in the DNA of rat aorta SMCs.
(c) In order to understand whether NO mediates the antiproliferative effect, we organized a series of experiments in which we co-incubated furoxans with 1H-[1,2,4]oxadiazole[4,3-α]quinoxalin-1-one (ODQ) and/or putrescine, thanks to their effect on the two known pathways by which NO exerts its antiproliferative effects on SMCs.
(d) Finally, we used a proteomic approach (SILAC, Western blot, and MetaCore) to find proteins targeted by furoxans and to unravel the pathways and molecular mechanism(s) underlying their pharmacological effect on SMC proliferation.

## 2. Results

### 2.1. Effect of Different NO Donors on NO Release, SMC Proliferation, and Possible Mechanisms

In a first attempt, we screened the antiproliferative ability of a library of classical NO donors, namely, mononitrates (compounds **1** and **3**) and dinitrates (compounds **2** and **4**; Table 1) in human SMCs.

As shown in Table 1, these compounds did not demonstrate significant antiproliferative properties in our experimental conditions, despite a relevant and significative vasodilating ability on isolated rat aorta strips [76]. Only compound **2** showed a mild effect (IC_50_ >100 µM).

Therefore, our attention moved to the analysis of the effects of differently substituted furoxans, whose general structure has been reported in Table 2.

Furoxans are known to differ in their reactivity towards thiol groups, in the kinetics, and in the extent of NO release. The reactivity towards thiols and the consequent NO release of selected furoxan derivatives (compounds **5** and **12**) was evaluated in a PBS solution in the presence of an excess of N-acetyl-L-cysteine. The reaction of N-acetyl-L-cysteine with furoxans is time and concentration dependent, as depicted in Figures S1 and S2 in the Supplementary Materials. In the same experiments, NO release was assessed by the detection of nitrite, the primary product of NO oxidation by O_2_ in aqueous solution using the Griess reaction. The formation of nitrite in these conditions is governed by a third-order rate law (Figures S1 and S2). Compound **5** showed a much higher reactivity than compound **12**, which is able to release twice the amount of NO compared to 5. We then analyzed the antiproliferative effect of a wide library of furoxan derivatives characterized by a variety of substituents at the 3/4 positions of the heteroring (compounds **5**–**19**, as shown in Table 2) in human SMCs, and we also showed that these products relaxed phenylephrine-precontracted rat aorta strips through a cGMP-dependent mechanism. This action was inhibited by ODQ, a well-known inhibitor of soluble guanylyl cyclase, thus reinforcing the involvement of NO in their vasodilating effect [76].

Groups in position 3 were selected in order to modulate the electrophilia of the same position. In detail, in blocking position 4 of the furoxan ring using a carbon phenyl group, the inhibitory potency on SMC proliferation paralleled with the different electron-withdrawing capacity of the group in position 3 of the ring (Table 2). 4-Phenylfuroxans, which bear an electron-withdrawing group in 3, were demonstrated to be the most potent, since the opening of the ring is favored.

To better understand the mechanism of action of furoxans on the inhibition of SMC proliferation, the study was also extended to 4-R_2_-3-phenylfuroxans, in which the groups in 3 and 4 were interchanged (molecules 15–19; Table 2) and to the structurally related furazans, devoid of the ability to release NO (i.e., molecules 20–27; Table 3).

**Figure 1:**
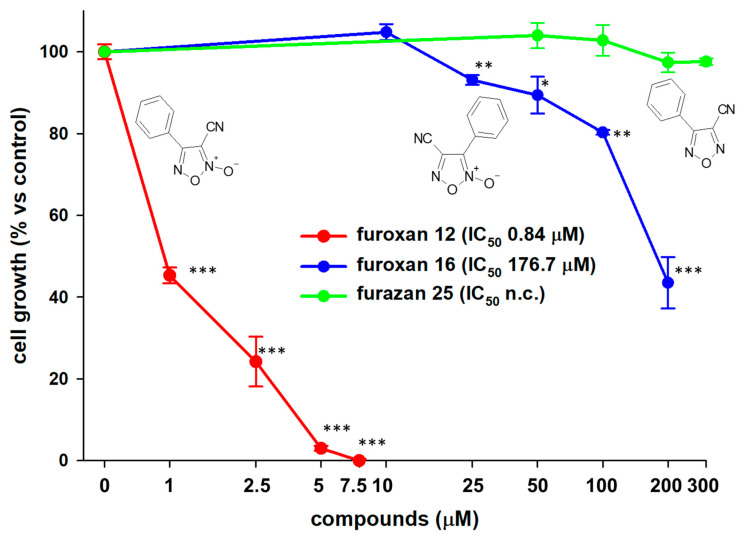
Effect of 3-cyano-4-phenylfuroxan (compound **12**), 4-cyano-3-phenylfuroxan (compound **16**), and their corresponding ineffective furazan (compound **25**) on proliferation of human SMCs. Cells were seeded at a density of 7 × 10^4^/Petri dish (35 mm) and incubated with MEM supplemented with 10% FCS. Twenty-four hours later, the medium was changed to one containing 0.4% FCS to stop cell growth, and the cultures were incubated for 72 h. At this time, the medium was replaced with one containing 10% FCS in the presence or absence (control) of known concentrations of the tested compounds, and the incubation was continued for a further 72 h at 37 °C. Cell proliferation was evaluated by cell count after trypsinization of the monolayers by a Coulter Counter model Z. Values are the mean ± SD of three different experiments, each run in triplicate * *p* < 0.05; ** *p* < 0.01; *** *p* < 0.001 vs. control (Student’s *t*-test).

When 3-cyano-4-phenylfuroxan (compound **12**) was tested together with its corresponding 4-cyano-3-phenylfuroxan (compound **16**) and 3-cyano-4-phenylfurazan (compound **25**) (Figure 1), compound **12** was demonstrated to be the most potent inhibitor of SMC proliferation, followed by the corresponding compound **16**, while compound **25** was not effective on this parameter, as evident from the respective IC_50_ values.

These results have been confirmed with other 3-phenyl-, 4-phenylfuroxans and their corresponding furazans, as shown in Table 2 and Table 3. The latter clearly demonstrates a gradual loss of potency both on SMC proliferation and vasodilation ranging from 4-phenylfuroxans to the corresponding 3-phenylfuroxans, up to the total ineffectiveness exerted by furazans. A detailed table on the effects of all the molecules we tested is reported in the Supplementary Materials (Table S1).

**Figure 2:**
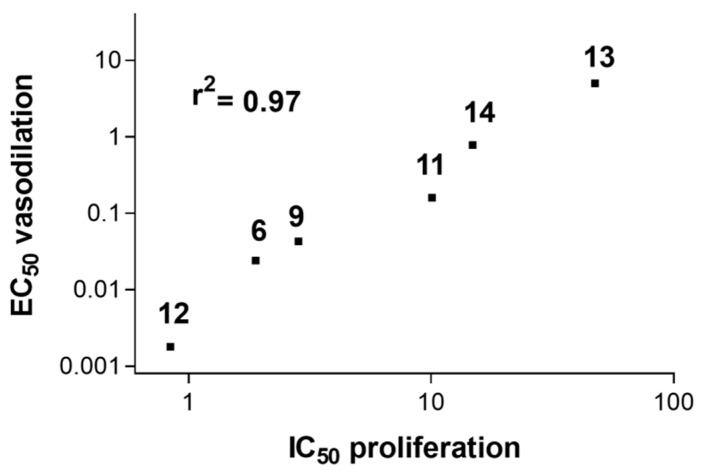
Linear relationship between the potency (µM) in inhibiting human SMC cell proliferation and vasodilating activity on rat aorta strips elicited by 4-Ph-furoxans with different substituents in position 3.

When the vasodilating ability on rat aorta strips (EC_50_) of different furoxans was plotted together with their antiproliferative effect (IC_50_) on human vascular SMCs, a linear correlation emerged (r^2^ = 0.97). This might suggest a common NO-mediated role between these two effects (Figure 2).

### 2.2. Cell-Phase-Specific Antiproliferative Effect of Furoxans

**Figure 3:**
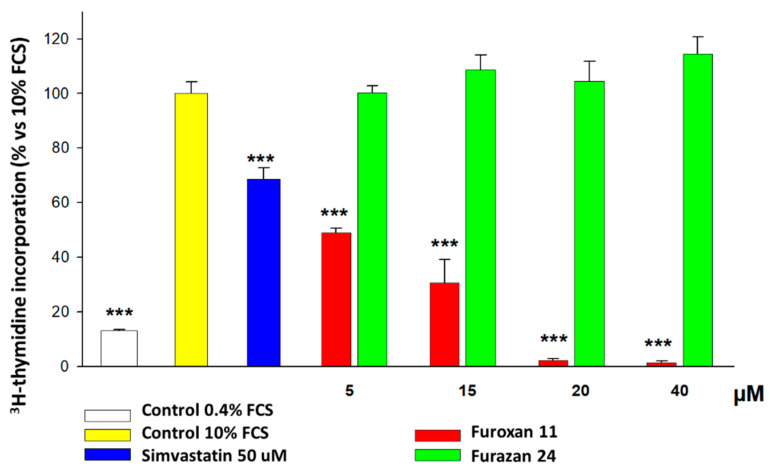
Effect of 3-acetoxy-4-phenylfuroxan (compound **11**) vs. its corresponding ineffective furazan (compound **24**) on thymidine incorporation in synchronized rat SMCs. Cells were seeded at 3 × 10^5^ cells/plate and synchronized by growing them for 120 h in a medium containing 0.4% FCS. Quiescent cells were then incubated for 20 h in fresh medium with 10% FCS, in the presence of the tested compounds. DNA synthesis was estimated by nuclear incorporation of [3H]-thymidine, incubated with cells (2 uCi /mL) for 2 h. Values are the mean ± SD of one experiment run in triplicate *** *p* < 0.001 vs. control (Student’s *t*-test).

In another set of experiments, we wanted to assess whether this antiproliferative effect could be specific to the cell-cycle phase; to achieve this aim, we utilized rat SMCs, thanks to their higher propensity to synchronize compared to human SMCs. After we confirmed that the potency of the molecules was almost identical between rat and human SMCs, we demonstrated that the antiproliferative effect is due to a specific inhibition of G1/S cell-cycle phase transition (Figure 3). In detail, the effect of furoxan 11 (IC_50_ 3.9 µM) was also confirmed with furoxan 6 (IC_50_ 1.5 µM) and with hybrids bearing an antioxidant and a furoxan moiety, but not by their related furazans (respectively 24 and 21), which were demonstrated to be ineffective in preventing SMC proliferation, even when this activity was measured by thymidine incorporation.

### 2.3. Effect of Intermediates of the Known NO-Dependent Pathways Regulating SMC Proliferation

**Figure 4:**
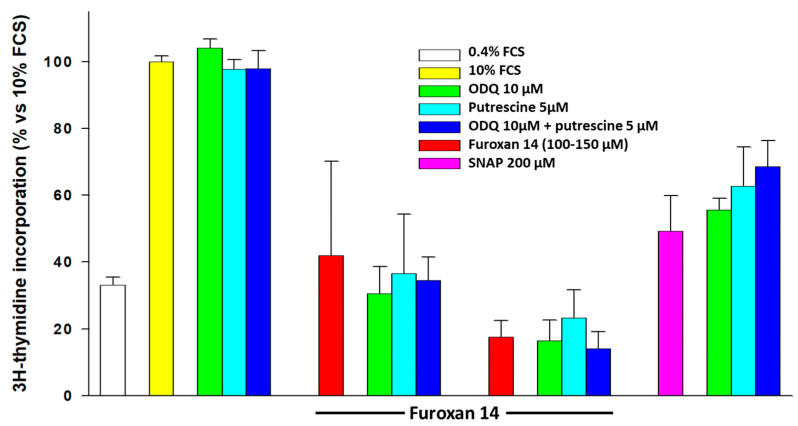
Effect of putrescine and ODQ, alone or associated, on thymidine incorporation in synchronized rat SMC treated with furoxan **14** or SNAP. Experimental conditions are the same as in Figure 3.

In order to understand whether the antiproliferative effect of furoxans on SMCs is dependent on NO release, we organized experiments in which these molecules were administered to SMCs together with inhibitors of the two well-known pathways (guanylyl cyclase and polyamines) by which NO exerts its antiproliferative effect. The soluble guanylyl cyclase inhibitor ODQ, added at least 30 min before the administration of furoxans (in order to deplete cells of cGMP), and putrescine, the product of ornithine decarboxylase, alone or together, were not able to prevent the inhibitory effect on SMC proliferation elicited by 3-carbamoyl-4 phenylfuroxan (compound **14**), evaluated as thymidine incorporation into newly synthesized DNA by rat SMCs (Figure 4). On the other hand, these compounds at least partially prevented the NO-dependent antiproliferative effect exerted by the classical NO-donor S-nitroso-N-acetylpenicillamine (SNAP) at high concentrations (200 µM).

### 2.4. Effect of Furoxans on Cellular Protein Expression by SILAC and MetaCore

**Figure 5:**
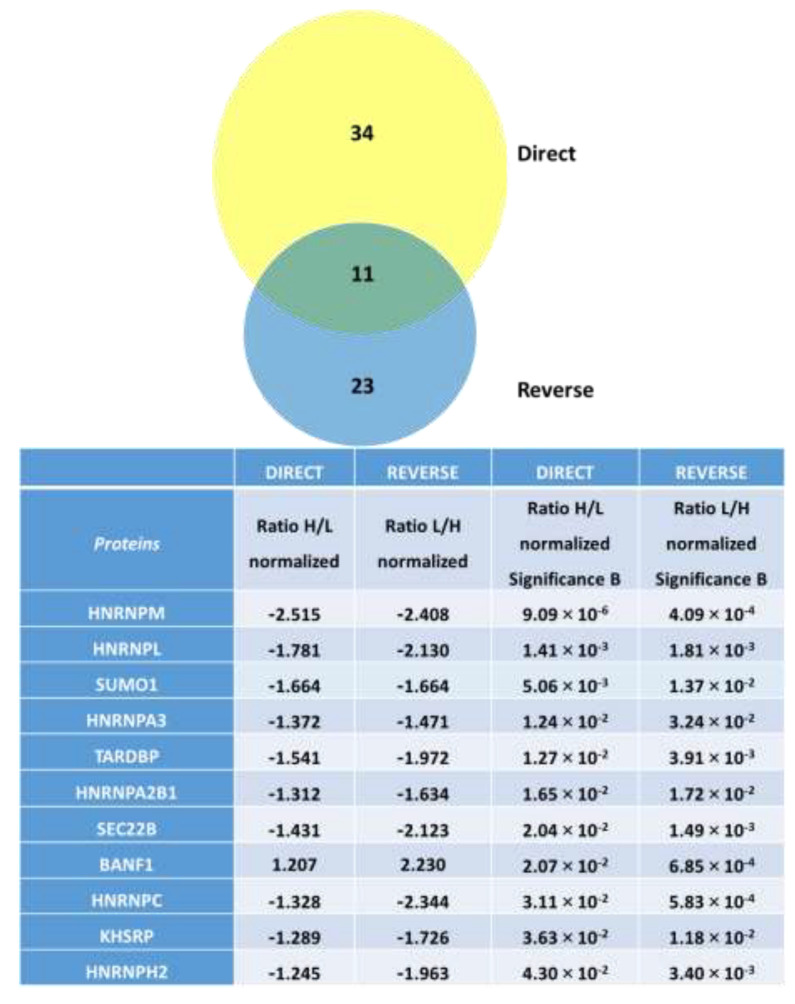
Upper panel: Venn diagram representing the 11 proteins modified by furoxan 12 as documented by SILAC technique. Lower panel: Table with ratios H/L, L/H, and significance B of the 11 modified proteins as found by direct and reverse experiments conducted by SILAC technique.

In order to understand the mechanism(s) underlying furoxans’ effect on SMC proliferation, we adopted a proteomic approach. In detail, in three different experiments, we treated SMCs with medium plus 10% FCS and vehicle (control) or with the 3-cyano-4-phenylfuroxan (compound **12**) under the same conditions utilized for the cell-cycle experiments, and we analyzed its pharmacological effect on the proteome using the SILAC technique. As evident from Figure 5, out of the 838 proteins detected by SILAC in our cell model, 45 and 34 were statistically modified in their abundance by furoxan 12, according to the results from the direct and reverse labeling experiments, respectively. Therefore, we performed a detailed analysis on those common eleven proteins significantly affected (FDR ≤ 0.05) in abundance (fold change ≥ 1.2) by the furoxan treatment (Figure 5) in both of the experiments (direct and reverse labeling).

With the exception of barrier-to-autointegration factor (BANF1/BAF), all of the identified proteins were downregulated by furoxan 12 and their absolute majority corresponds to RNA binding proteins (RBP), in particular to heterogeneous nuclear ribonucleoproteins (hnRNPs). The ubiquitin-like protein small ubiquitin-related modifier 1 (SUMO1) and the vesicle-trafficking protein SEC22b (SEC22B) are the only two non-RBP proteins downregulated by the furoxan.

**Figure 6:**
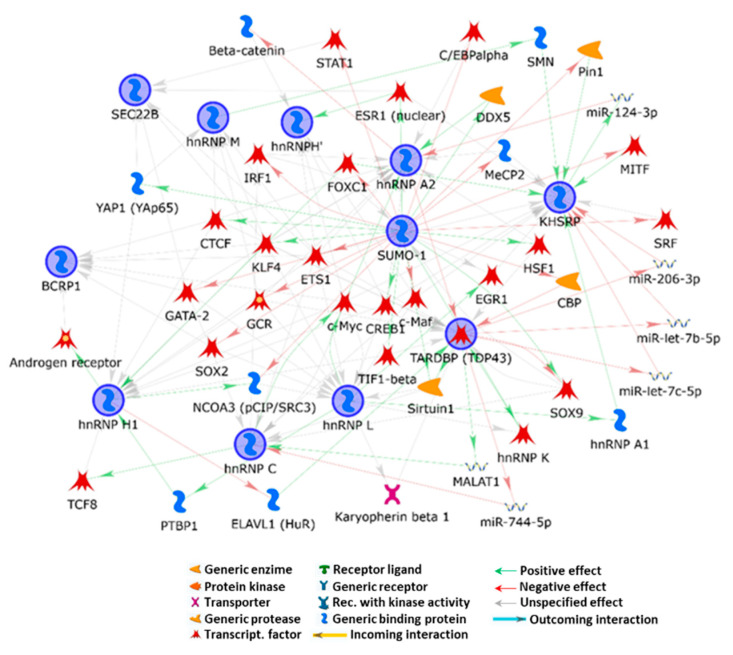
MetaCore network analysis of the related molecular processes and biochemical pathways underlined by their functional cooperation in furoxans’ antiproliferative effect on SMCs. SUMO1 appears as the central hub.

In order to evaluate the functional relevance of these proteins, the related molecular processes and the biochemical pathways underlined by their functional cooperation in furoxan’s inhibitory effect on SMCs, we performed MetaCore network analysis by applying the shortest path algorithm of the network building tool. Despite their small number, all of the experimental co-processed proteins entered the network (Figure 6).

This result corroborates the quality and relevance of the SILAC analysis in acquiring the “core proteins” of the furoxan-induced effects in SMCs. The built net actually highlights a tight functional correlation between the identified proteins and recognizes SUMO1 as the central hub. SUMO1 is a member of the SUMO family involved in post-translational modifications regulating DNA replication, transcription, chromatin organization, cell cycle, sub-cellular localization, protein–protein and protein–DNA interactions, and DNA damage response and degradation [84,85,86,87,88], and also plays important roles in atherosclerosis and vascular SMC proliferation (see Discussion section). Through numerous interactions with different proteins (mainly transcription factors added by the software), SUMO1 indirectly correlates to all of the other proteins deregulated by the treatment. In all cases, its mediated cross-linking to another experimental protein occurs by several factors added by MetaCore, thus suggesting SUMO1 is a multilevel controller of the biological functions exerted by the furoxan-modulated proteins.

Interestingly, a number of the proteins and non-coding RNA used by the software to cross-link experimental proteins are known to be involved in a wide plethora of atherosclerosis processes. Among others, SOX9 [89], NF-kB [90,91], HSF1 [92], EGR1 [93], c-Maf [94], IRF1 [95], ETS1 [91], CTCF [96], STAT1 [97], and SRF [98] actually impact SMC proliferation, extracellular matrix composition and calcium deposition, cytokine synthesis and secretion, lipogenesis and cholesterol metabolism, inflammation, senescence and apoptosis, and plaque onset and evolution. Furthermore, their functions are emerging as being strongly related to various non-coding RNA with critical activity in cardiovascular disorders. Several of the above-reported transcription factors have been also proposed or are already in use as targets in atherosclerosis therapy.

The functional overview of our results suggests a furoxan-induced generalized destabilization in the metabolism of coding/non-coding RNA by RBPs, and in proteostasis by RBPs, SUMO, and SEC22B that develops in decreased SMC proliferation. Nonetheless, by evaluating the functions and properties of the proteins modulated by the furoxan treatment, the reduced cell cycle rate we described could not be the result of a senescence process, which may negatively impact in SMC activities, but rather by a fine balance between pro- and anti-apoptotic factors, which leads to a reduced proliferation rate.

**Figure 7:**
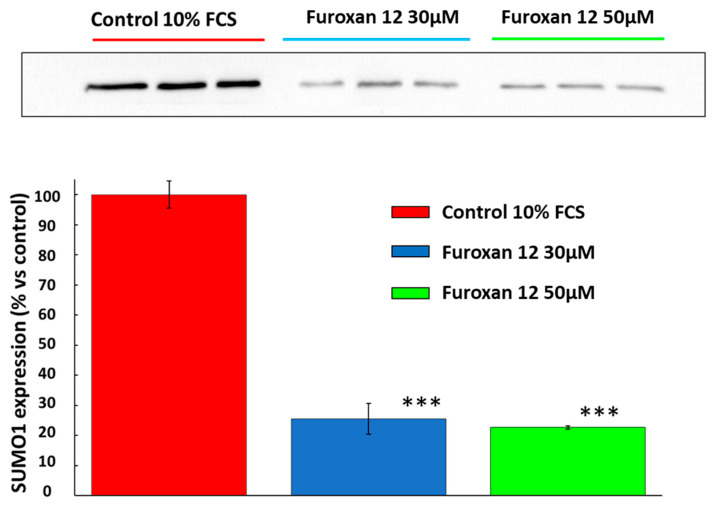
Western blot and densitometric analysis of the effect of furoxan 12 on SUMO1 expression in SMCs. *** *p* < 0.001 vs. control (Student’s *t*-test).

To confirm the results obtained by SILAC, which highlight the importance of SUMO1 as a central player of furoxans’ effect on SMC proliferation, we conducted Western blot experiments on SMCs treated with 10% FCS plus vehicle (control cells) or with the same furoxan utilized for SILAC experiments (e.g., furoxan 12). As shown in Figure 7, after 20 h of incubation, furoxan 12 significantly and concentration-dependently (up to 75%) decreased SUMO1 expression compared to the control, corroborating the indications for a role for SUMO1 in furoxans’ antiproliferative effect.

## 3. Discussion

Vascular hemodynamic and structural factors contribute to plaque formation and development, with SMC proliferation playing an essential role, especially in early atherogenesis, while the same process seems to prevent the rupture of the fibrous cap in advanced plaques [99]. This dilemma has always divided scientists on the relevance and the necessity of inhibiting SMC proliferation as an antiatherosclerotic approach. In the past few years, genetic lineage tracing studies showed that plaque SMCs are inhomogeneous and that simultaneous processes occurring in the plaque complicate the dissection of the role of SMCs in atherosclerosis [20,22,99,100]. Indeed, the balance between proliferation/migration and death/senescence is a key determinant of SMCs in the plaque [101]. For example, SMC apoptosis is low in early lesions, while it increases in advanced ones, particularly in necrotic core and fibrous cap [101,102,103]. It is noteworthy to underline that, since plaque rupture mostly occurs in the shoulder enriched in macrophages rather than SMCs, SMC apoptosis and inflammation may affect vulnerable plaque characteristics, accelerate lesion progression, and promote calcification [99,104,105]. In fact, aged SMCs present an increased expression of IL-6, chemokines, adhesion molecules, and Toll-like receptor 4, suggesting a proinflammatory environment. Therefore, since SMC phenotypic switching, death, and senescence are promoters of inflammation, monocyte recruitment and the secretion of SMC mitogens, reducing SMC proliferation, may result in beneficial vascular effects [63].

In the present work, we demonstrated that, according to the experimental conditions applied, furoxans concentration-dependently inhibit SMC proliferation, albeit with significant differences in potency (Table 2 and Table 3), thanks to the modulation of the effect achieved by changing the nature of substituents in position 3 of the ring. Furoxans’ antiproliferative effect is evident at concentrations lower than those effective after the administration of classic NO donors such as mono- and dinitrate esters, SNAP, molsidomine, and linsidomine, suggesting that, if NO is the culprit of the effect, a different modality or a better compartmentalization of NO release may be involved [82].

One of the most used clinical approaches to restore blood flow in stenotic vessels is the implantation of stents. Nevertheless, a significant number of interventions fail due to restenosis caused by extensive SMC proliferation after the alteration of vessel integrity. NO donors/mimetics showed vascular protection, at least in rat models of restenosis, by inhibiting SMC migration and proliferation [68,106,107]. A device producing NO long term in a cGMP-dependent fashion developed by Yang et al. [68] reduced platelet activation and adhesion, and the proliferation and migration of SMC, while enhancing endothelial cell migration and growth. When implanted in rabbit arteries, it promoted re-endothelization and reduced the risk of restenosis [94]. Therefore, delivery stents medicated with furoxans may be useful in patients after coronary stent implantation or aortic bypass thanks to their favorable pharmacokinetics and pharmacodynamics. 

To demonstrate whether the ring opening and the subsequent NO release by furoxans could be responsible for their antiproliferative effect on SMC, we also utilized furazans, compounds similar to furoxans from a chemical/physical point of view, but devoid of the ability to release NO. As shown in Table 3, the opening of the ring is essential for the antiproliferative effect exerted by furoxans. Moreover, when utilizing furoxans in which the groups in position 3 and 4 are exchanged, 3-phenyl-furoxans are still able to inhibit cell proliferation, but at higher concentrations. This evidence is in line with the results demonstrated by Gasco et al. [75], who reported a lower potency in the vasodilating ability of rat isolated aorta rings by 3-phenyl furoxans when compared to corresponding 4-phenyl furoxans. In particular, an electron-withdrawing group in position 3 favors furoxan ring opening and, consequently, NO release. This property is very useful from a pharmacological point of view, since the release of NO may be modulated depending on the needs. For example, the 3-cyano-4-phenylfuroxan may be utilized if a “strong” and immediate effect is requested, while other molecules characterized by a sustained and time-controlled release may be optimal in case of chronic pathologies such as atherosclerosis.

When searching for a cell cycle phase specific effect, we found a block of thymidine incorporation exerted by 4-phenylfuroxan, but not by furazans, suggesting that active compounds inhibit cell proliferation due to a specific G1-S arrest. It has to be highlighted that when furoxans have been administered for no longer than 20 h, to elicit a significant effect, their concentrations have been increased vs. cell counting experiments performed after 72 h of incubation. These data have been confirmed by cytofluorimetric analysis. Nevertheless, this effect is also shared by NO [83,108].

Altogether, in order to understand whether the antiproliferative effect on SMC is NO-mediated, based on the aforementioned experimental evidence, we could not rule out its contribution, since we demonstrated:

(a) That upon thiol-mediated activation, furoxans time- and dose-dependently generate NO (Figures S1 and S2);
(b) The ability of all furoxans, despite different potencies, but not of furazans, to inhibit SMC proliferation;
(c) A significant strong (r^2^ = 0.97) direct correlation (Figure 2) between the reduction in SMC growth and a NO-dependent vasodilating effect on isolated rat aorta stripes exerted by furoxans;
(d) That the G1-S phase-specific antiproliferative effect is shared by furoxans and NO [62,63].

To settle the question of the direct involvement of NO on furoxans’ inhibition of SMC proliferation, we administered cells with furoxans plus:

(a) The inhibitor of soluble guanylyl cyclase, ODQ, to deplete cells from cGMP;
(b) Putrescine, a product of ornithine decarboxylase, the enzyme inhibited by NO eventually released by furoxans;
(c) Their association.

Our data clearly show that neither putrescine nor ODQ (alone or associated) prevented the furoxan-mediated inhibition of SMC proliferation, differently from what was observed after administration of the classical NO-donor SNAP. The fact that NO released in the culture medium does not correlate with furoxans’ potency in inhibiting SMC proliferation, differently from what happens after SNAP, together with these data suggest that, despite NO release after furoxan ring opening being a necessary condition, NO is not the real effector of the antiproliferative effect.

A probable and so far unknown reasonable alternative mechanism, consisting of the S-nitrosylation of a protein involved in the proliferative process, has been proposed to explain the capacity of furoxans to inhibit thioredoxin glutathione reductase, an enzyme required by Schistosoma to maintain proper cellular red-ox balance [109,110]. Schistosomiasis is an endemic illness in Africa, southern America, and south-eastern Asia for which only praziquantel therapy is available [111]. Despite the most potent furoxans we tested in our cell model being the most potent in killing schistosomes [110], we could not demonstrate any presence of S-nitrosylated proteins involved in cell proliferation after furoxan treatment.

Altogether and based on our data, we propose that the opening of the furoxan ring by thiols, besides releasing NO, generates a series of intermediates that may interact with specific cellular targets by the formation of covalent or reversible bonds responsible for their antiproliferative effect.

According to our proteomics results, hereafter, we discuss the properties of furoxan-affected proteins and we reason on how they may modulate SMC behavior, as well as on how their expression changes may impact on in vivo SMC phenotypes and in atherosclerosis onset and development. Our discussion on these proteins aims to evaluate the reliability in proposing further investigations on furoxan use for atherosclerosis treatment.

### 3.1. Small Ubiquitin-Related Modifier 1 (SUMO-1)

SUMO1 is a protein of the SUMO family that plays important roles in cell homeostasis and proliferation [84,112]. Moreover, we found a significant reduction in its expression after furoxan treatment, suggesting its involvement in the antiproliferative effect exerted by furoxans. Indeed, the role of SUMO in atherosclerosis is well-exemplified by the reduction in NO generation due to SUMOylation of the proatherogenic activator of transcription factor 3 (ATF3) or by its modulating properties on NLRP3 inflammasome activity, release of inflammatory cytokines, and expression of adherence molecules [84,113]. Indeed, the SUMOylation of the antiatherogenic extracellular signal-regulated kinase 5 (ERK5) accelerates inflammation and increases VCAM-1 and ICAM-1 expression. ERK5 activation also upregulates KLF2 and KLF4, which also reduce inflammation, but its SUMOylation due to turbulent flow increases ROS production, thus blocking its atheroprotective role [114]. Also, p53 SUMOylation induced by protein kinase Cζ leads to endothelial cell apoptosis and accelerated atherosclerosis. The dyslipidemic effects of SUMOylation are related to a decrease in PPARα transcription and of LDL-receptor expression via sterol regulatory element-binding protein 2 (SREBP2) [84,113,114]. Finally, PPARγ SUMOylation suppresses its transcriptional activity, thus inducing dyslipidemia and vascular SMC proliferation [114].

SUMOylation interferes with many pathways involved in vascular SMC proliferation. In fact, Ang II/AT1 receptor-mediated SMC proliferation positively correlates with Rho-specific guanine nucleotide dissociation inhibitor RhoGDI1 SUMOylation. Moreover, PDGF-BB promotes KLF4 SUMOylation, which acts as a switch in transcriptional programs controlling SMC proliferation, also by reducing p21. Interestingly, SUMOylated KLF4 does not affect KLF4 expression, thereby forming a positive feedback loop enhancing proliferation, demonstrating that SUMOylated KLF4 reverses the transactivation action of KLF4 on p21 induced by PDGF-BB [84,86,113,115,116]. Finally, the high expression of SUMO1-induced SUMOylation of vacuolar protein sorting 34 (VPS34), which mediates the effects of hypoxia on VSMC proliferation, promotes the assembly of the Beclin-1-Vps34-Atg14 complex and autophagic activation [117].

### 3.2. BANF1 and Vascular SMC Mechanical Stress

Blood vessels are subjected to pressure and flow mechanical loads. Vascular endothelial cells and SMCs perceive, through mechanosignaling, these stimuli and respond by modulating gene expression, cellular morphology, and function [118]. Aberrant mechanical forces typical of atherosclerosis and hypertension may, therefore, cause vessel remodeling and dysfunctions [119]. Mechanosignaling propagates throughout the ECM to SMC sensing membrane proteins and structures up to the nucleus membrane by a complex cytoskeleton protein framework, and, finally, to the DNA through the linker of nucleoskeleton and cytoskeleton complex (LINC) and nuclear lamina [118].

The barrier-to-autointegration factor (BANF1/BAF; BCRP1 in DIN) is a highly conserved DNA-binding protein that interacts with LEM-domain proteins, several of which are anchored to the inner nuclear membrane, such as the LINC protein emerin [120]. BANF1 is a component of the nuclear lamina structure that directly transduces mechanical signals to the genome by tethering LINC and lamins, through histones H4, H3, and H1.1, to DNA [121,122]. BANF1 influences the higher-order chromatin structure [123] to modulate gene expression and epigenetic regulation, mainly by reducing histone acetylation [120]. Recently, the emerin-dependent re-localization of BANF1 during early phases of oxidative stress has also been suggested to occur in cellular process modulation to efficiently counteract the oxidative stress [124].

Despite its upregulation is often associated with cancer development and cancer cell proliferation and migration [125,126], in in vivo SMCs, BANF1 upregulation might increase resistance to vessel mechanical stress by playing a critical role in nuclear rupture repair [127]. Transient nuclear envelope ruptures can lead to DNA damage, which is directly related to aging and, eventually, to apoptosis. Since reducing the death of foam cells and SMCs is a therapeutic goal to prevent atherosclerosis development and worsening and to stabilize the lesion cap [128,129], in vivo BANF1 upregulation could have a role in decelerating foam cells and SMC death and in reducing plaque instability. Moreover, BANF1 has recently been proved to be a regulating double-strand break repair pathway, by modulating DNA-PKC activity [130] and oxidative-stress-induced DNA damage [131]. As a rule, BANF1 control on genome stability and heterochromatin maintenance may also reduce altered transcription and cellular senescence [130]. Its correlation with aging and nuclear stability is further stressed by the Nestor–Guillermo progeria syndrome (#614008), an autosomal recessive disorder caused by BANF1 mutation, that is characterized by low BANF1 levels and its reduced ability to bind DNA [132,133].

In addition, this nuclear lamina component could increase SMC tolerance to mechanical injuries and related signaling, hence reducing the VSMC contractile phenotype switch to the synthetic one. Although lamin A expression is reduced under pathological cyclic stretch, and despite this results in increased SMC proliferation [118], raised BANF1 concentrations could strengthen heterochromatin integrity by anchoring it to the nuclear lamina, regardless of lamin A presence, via BANF1 binding to other LEM-domain proteins or by counteracting nuclear ruptures, thus reducing SMC proliferation. Therefore, the furoxan-induced BANF1 increase may concur to the inhibition of SMC proliferation we documented.

### 3.3. Furoxan Modulation of Coding and Non-Coding RNA-Binding Proteins and VSMC Proliferation

RNA-binding proteins (RBPs), and, in particular, heterogeneous nuclear ribonucleoproteins (hnRNPs), are the main class of proteins we detected as being significantly downregulated in VSMCs treated with furoxans. While BANF1 may transduce mechanical and oxidative stress signals to the genome and interfere in gene expression by acting on histones and DNA integrity, hnRNPs regulate gene expression and related cellular activities mainly by controlling the processing of coding and non-coding RNA. Differentially occurring hnRNPs impact on the proteoform expression pattern of a cell by cooperating to modulate the properties and functions of different spliceosomal complexes [134]. Hence, the hnRNP abundance differences we observed may be considered the furoxan 12 spliceosome signature in in vitro SMCs and the reduced proliferation rate of SMCs we observed could be, at least in part, an effect of such a signature.

Besides their critical role in heterogeneous nuclear ribonucleoprotein complexes, numerous hnRNPs have also been described as having functions unrelated to RNA metabolism and whose deregulation correlates to various disorders [135]. In particular, increasing attention is drawn to the emerging role they exert by forming functional complexes with non-coding RNA in inflammation and immune response as well as in apoptosis and cell proliferation [136,137,138]. Recently, hnRNPs have been also proposed to operate in association with lncRNA as regulators of cellular processes in atherosclerosis [139].

In our study, hnRNPM is the most downregulated protein with the highest significant value of FDR due to the furoxan exposure (Figure 6). This RBP, previously described in cancer metastasis and muscle differentiation [140], exerts a key role in modulating macrophage transcriptomes for differential proteoform expression in innate immune responses. In particular, hnRNPM has been reported to induce cytokine production, e.g., in Kupffer cells, and to regulate anoikis in cancer [141]. Since SMCs were shown to differentiate towards a macrophage-like phenotype in a mouse model of atherosclerosis and they are known as being active in proinflammatory cytokine production and secretion [142,143], we may hypothesize a hnRNPM activity in SMCs similar to that described in macrophages.

Although a functional correlation has not yet been described in SMCs between them, hnRNPM was reported to upregulate the expression of the lncRNA nuclear paraspeckle assembly transcript 1 (NEAT1) in neuronal cells, and NEAT1 expression levels have been proved to control the SMC phenotype [144,145]. While its downregulation correlates with the expression of SMC genes and reduced proliferation and migration, NEAT1 upregulation causes the SMC switch from the quiescent contractile phenotype to the synthetic migratory one. The furoxan-induced depletion of hnRNPM concentrations in VSMCs may genuinely reduce NEAT1 stability and SMC proliferation, with a conceivable in vivo decrease in SMCs’ contribution to atherosclerosis.

As in hnRNPM, hnRNPL is overexpressed in several tumors where it controls cell proliferation, survival, and invasion [146]. In macrophages, hnRNPL was proved to form a functional complex with the THRIL (TNFα- and hnRNPL-related immunoregulatory lincRNA) lincRNA (long intergenic noncoding RNA) that controls TNFα expression [147]. It was also described as active in integrin and ECM protein synthesis during epidermal renewal and in an increase in ECM stiffness in idiopathic pulmonary fibrosis [135,148]. Furthermore, hnRNPL controls VEGF expression in myeloid cells by stabilizing its messenger in response to hypoxic stress [149]. In atherosclerotic lesions, hypoxia and inflammation are known to induce SMCs and macrophages to produce VEGF-A that stimulates intima-SMC and blood monocyte migration into the plaque and, probably, SMC proliferation [150,151,152].

Although its role has to be defined in SMC phenotype determination and atherosclerosis development, the hnRNPL decrease may concur, according to the several processes it modulates in different cell types, to the cytostatic effect of furoxan treatment on in vitro SMCs. In addition, we may speculate that in atherosclerosis hnRNPL downregulation may have a positive effect in reducing inflammation and the accumulation of cells and extracellular matrix between the vessel endothelium and tunica media. 

Also, FBP2 (KHSRP in MetaCore), a member of the single-stranded DNA-binding protein family, is a furoxan-affected RBP that was found overexpressed in different cancers and that, depending on the tumor cell type, is involved in cell proliferation, migration, and drug resistance [153,154,155,156,157]. Besides its direct role in DNA transcription, FBP2 is also critically active in the regulation of mRNA and miRNA maturation and transport as well as in AU-rich element (ARE)-directed mRNA decay [157,158]. Since FBP2 was described mediating G1/S transition by suppressing CDKN1a mRNA expression [159,160], the furoxan 12-induced decrease in this protein may concur to the VSMCs’ reduced proliferation. This data is corroborated by the G1/S phase arrest we observed in furoxan 12-exposed SMCs.

The H_2_O_2_-responsive protein hnRNPC is another furoxan-downregulated protein (Figure 6) critically active in cell proliferation and differentiation. Noteworthy, its expression was found consistently upregulated in intimal and medial SMCs from pre-atherosclerotic hyperplastic intima and atherosclerotic lesions, respectively [161]. In fact, it facilitates the expression of cell growth- and survival-regulating proteins (e.g., platelet-derived growth factor PDGF, c-myc, and X chromosome-linked inhibitor of apoptosis) and it was proposed as an indicator of SMC activation [161]. Similarly to hnRNPM, its downregulation may be of relevance in reducing SMC proliferation in vitro, while in vivo, it may delay the SMC switch from a contractile to synthetic phenotype.

Furthermore, along with the furoxan 12-decreased hnRNPA2B1 (Figure 6) previously reported as augmented in atherosclerotic plaques [162], hnRNPC is a nuclear reader of methylation at the N-6th position of RNA adenosine residue (m6A). This epitranscriptomic modification of coding and non-coding RNAs influences RNA stability, functional specificity, localization, splicing and translation (for mRNA), and degradation [163]. Since m6A increases RNA accessibility to hnRNP binding, variations in m6A levels may impact, directly or indirectly, on the transcriptome. Notably, deregulation in adenosine methyltransferases (writers), demethylating enzymes (erasers), and m6A-binding proteins (readers) as well as m6A increase have been described in atherosclerotic plaques and CV diseases and affecting vascular inflammation and cholesterol metabolism during atherosclerosis [148,163,164,165]. We may, hence, hypothesize that hnRNPC and hnRNPA2B1 downregulation could reduce the deleterious effects that m6A increase has in atherosclerosis RNA metabolism and alternative maturation. To further stress the relevance of hnRNPA2B1 decrease in protecting against atherosclerosis-related affections, Zhang et al. proved that lncRNA AC105942.1 suppresses SMC pathological proliferation by downregulating hnRNPA2B1 and suggested the latter as a target for novel therapeutic attempts [162,166]. As it is known, mitochondrial defects are widely associated with oxidative stress, dysfunctions in lipid metabolism, calcium homoeostasis, and inflammatory response, all processes occurring during atherosclerosis onset and development [167,168,169]. TAR DNA-binding protein 43 (TDP-43), coded by the *TARDBP* gene, is a member of the hnRNP family involved in mitochondrial dynamics and aggregation, and whose upregulation and cytoplasmic accumulation were associated with mitophagy, mitochondrial fragmentation, and apoptosis [170,171,172,173]. As suggested by its correlation with different miRNAs and with the MALAT1 lncRNA [174] in the shortest path network, TDP-43 is involved in several processes related to nuclear and mitochondrial gene expression as well as to lipid and glucose metabolism [170,172,175,176] by regulating transcription, RNA maturation, and mRNA stability. TDP-43 downregulation may protect SMCs from apoptosis or senescent phenotype acquisition, probably also in in vivo stressed vessels.

On the other hand, hnRNPA3, which plays a key role in the fibroblast senescence spliceosome [134], destabilizes the cyclin-dependent kinase inhibitor 2A (p16-INK4), a redox-imbalance activated factor that is involved in senescence-associated growth arrest [177]. Although the senescence phenotype may have deleterious effects on atherosclerosis onset and development, a hnRNPA3 decrease may simply limit SMC proliferation and, in vivo, it may antagonize an environment favoring SMCs to acquire the synthetic migratory phenotype. While TDP-43 regulates the majority of the SPN-reported miRNAs and the MALAT1 lncRNA, FBP2 and hnRNPC are differentially targeted by the SPN miRNAs controlled by TDP-43 (i.e., miR-let-7c-5p, miR-let-7b-5p, miR-744-5p, and miR-206-3p) and by MALAT1. In addition, FBP2 modulates miR-124-3p activity that, in turn, regulates hnRNPA2B1 (Figure 7). Taken together, these RBP/RNA cross-talks suggest an interesting tight interaction niche among different RBPs and non-coding RNAs that correlate with the furoxan 12 spliceosome signature and that may cooperatively affect SMCs’ properties by changing their RNA and protein profiles.

Furthermore, in atherosclerosis and related morbidities, an increasing body of evidence suggests that oxidized LDL may regulate the SMC proliferation/apoptosis balance through miRNAs, as was recently proven for the miR-124-3p/DLX5 axis. This FBP2 positively controlled miRNA inversely correlates with collagen plaque content and plaque stability in ApoE−/− mice and was recently reported to inhibit proliferation and increasing apoptosis in SMCs [178,179]. Ox-LDL, by inducing the downregulation of miR-124-3p, causes SMC hyperproliferation. In addition, miR-124-3p is also known to affect SMC proliferation and migration by interfering with STAT3 mRNA and protein levels [180].

### 3.4. Furoxan Treatment Implications in Vesicle Trafficking

The downregulation of the vesicle-trafficking protein SEC22 homolog B (SEC22B) (Figure 6) suggested an SMC response to furoxan 12 treatment involving the soluble N-ethylmaleimide-sensitive factor attachment protein receptors (SNAREs). SEC22B is a key factor in vesicle fusion to target membrane during the anterograde and retrograde vesicular trafficking between the ER and the Golgi apparatus. This R-SNARE component was also associated with phagocytosis and with autophagosome formation and fusion with lysosome or, in secretory autophagy, with plasma membrane [181]. Of note is that the atherosclerotic critical cytokine interleukin-1β (IL-1β) can be released through secretory autophagy and SEC22B is crucial for the process, as it was proven by the IL-1β secretion decrease as a consequence of SEC22B downregulation [182,183,184,185,186]. We suppose that SEC22B downregulation can simply play a role in diminishing the proliferation of furoxan-treated SMCs according to its involvement in the cell cycle and tumorigenesis [187].

## 4. Materials and Methods

### 4.1. Chemicals

Eagle’s Minimum Essential Medium (MEM), trypsin ethylendiaminetetraacetate, penicillin (10,000 U/mL), streptomycin (10 mg/mL), nonessential amino acid solution (100×), fetal calf serum (FCS), disposable culture flasks, Petri dishes (Corning), and filters (Millipore) were purchased from Euroclone (Milano, Italy). [6–3H]-thymidine, sodium salt (2 Ci/mmol) and molecular-weight protein standards were from Amersham. Isoton II was purchased from Instrumentation Laboratories (Milano, Italy). Sodium dodecyl sulfate (SDS), NNNN-tetra-methyl-ethylendiamine, ammonium persulfate, glycine, and acrylamide solution (30% T, 2.6%) were obtained from Bio-Rad Laboratories. Simvastatin in its lactone form (Merck, Sharp, & Dohme Research Laboratories, Rahway, NJ, USA) was dissolved in 0.1 M NaOH to give the active form, and the pH was adjusted to 7.4 by adding 0.1 M HCl. The solution was sterilized by filtration.

### 4.2. Preparation of the Study Compounds

Furoxans and related molecules were kept at 4 °C and in the dark until use. Fresh DMSO solutions were prepared for each experiment. Simvastatin (sterile aqueous solutions) was utilized as a positive control as an antiproliferative agent, as previously demonstrated by our group [188,189,190]. When used, the inhibitor of the soluble guanylyl cyclase (1H-[1,2,4]oxadiazole [4,3-alph19uinoxalinelin-1-one (ODQ)) was administered to cells at least 30 min prior to the compounds being assayed in order to deplete cell cGMP.

### 4.3. Reactivity and NO Release of 3-cyano-4-phenyl Furoxan (Compound ***12***) and 3-phenylsulfonyl-4-ethoxy Furoxan (Compound ***5***)

Compounds **12** and **5** were dissolved in PBS (0.05 M, pH = 7.4, 1% DMSO) at 0.1 mM concentration; compound **12** was incubated at 37 °C in the presence of N-acetyl L-cysteine 0.5 mM (5×), 5 mM (50×), 0.1 M (1000×) for 5 h; compound **5** was incubated at 37 °C in the presence of N-acetyl L-cysteine 0.5 mM (5×) and 5 mM (50×) for 2 h. Each experiment was conducted in triplicate. At appropriate time intervals, the reaction mixture was analyzed by HPLC to quantify the remaining compound and by Griess assay to quantify nitrite (the main product deriving from NO in solution in the presence of O_2_). RP-HPLC analyses were performed on an HP1100 chromatograph system (Agilent Technologies, Palo Alto, CA, USA) equipped with a quaternary pump (model G1311A), a membrane degasser (G1379A), and a diode-array detector (DAD) (model G1315B), integrated into the HP1100 system. Data analyses were processed using an HP ChemStation system (Agilent Technologies). The analytical column was a Zorbax Eclipse XDB-C18 (4.6 × 150 mm, 5 µm, Agilent Technologies). The mobile phase consisted of acetonitrile 0.1% TFA/water 0.1% TFA 50/50 *v*/*v* at flow-rate = 1.0 mL/min. The injection volume was 20 µL (Rheodyne, Cotati, CA, USA). The column effluent was monitored at 254 nm, referenced against a 700 nm wavelength. Compound quantification was carried out using calibration curves obtained by analyzing five standard solutions of the compounds solubilized in eluent (linearity determined in a concentration range of 1–100 µM; r^2^ > 0.99). For the determination of the nitrite produced, 1 mL of the reaction mixture was treated with 250 µL of the Griess reagent (sulphanilamide (4 g), N-naphthylethylenediamine dihydrochloride (0.2 g), and 85% phosphoric acid (10 mL) in distilled water (100 mL final volume)), and after 10 min at room temperature, the absorbance was measured at 540 nm (spectrophotometer UV-2501PC, Shimadzu); sodium nitrite was used for the calibration curve (seven standard solutions at concentrations between 10 and 60 nmol/mL, r^2^ > 0.99). The yield in nitrite is expressed as percent NO_2_^-^ vs. incubated compound (mol/mol) ± SE.

### 4.4. Cells and Cellular Protocols

Human (A617 line, from femoral artery) and rat SMCs cultured from the intimal-medial layers of the aorta of male Sprague Dawley rats were seeded at a density of 7 × 10^4^ or 2 × 10^5^ SMC/Petri dish (35 mm), respectively, and incubated with MEM supplemented with 10% FCS. Twenty-four hours later, the medium was changed to one containing 0.4% FCS to stop cell growth and the cultures incubated for 72 h. At this time (time 0), the medium was replaced with one containing 10% FCS in the presence or absence of known concentrations of the tested compounds and the incubation was continued for a further 72 h at 37 °C. At the same time, just before the addition of the substances to be tested, three Petri dishes were used for cell counting and the obtained mean value subtracted from the cell number at the end of the treatment to give the number of cells grown in the presence of the pharmacological treatment. Cell proliferation was evaluated by cell count after trypsinization of the monolayers by a Coulter Counter model Z (Beckman Coulter, Milano, Italy) [188,189].

In a subsequent set of experiments, the synchronization of rat SMC to the G0/G1 interphase of the cell cycle was accomplished by incubating logarithmically growing cultures (3 × 10^5^ cells/plate) for 120 h in a medium containing 0.4% FCS. Quiescent cells were incubated for 20 h in a fresh medium with 10% FCS in the presence of the tested compounds. DNA synthesis was then estimated by nuclear incorporation of [3H]-thymidine, incubated with cells (2 uCi /mL) for 2 h, as previously described [188,190].

### 4.5. Electrophoresis and Western Blotting

The cells were washed twice with PBS and lysed by incubation with a solution of 50 mM Tris pH 7.5, 150 mM NaCl, 0.5% Nonidet-P40, containing protease and phosphatase inhibitor cocktails (Merck KGaA, Milan, Italy) for 30 min on ice. The cell lysates were then cleared by centrifugation at 14,000 g for 10 min and protein concentrations determined using the BCA protein assay (Pierce, Rockford, IL, USA). Twenty micrograms of total protein per sample was separated by a 12.5% SDS-PAGE under reducing conditions. After a two-hour run, the proteins were transferred overnight on a nitrocellulose membrane (Millipore) and subsequently immunoblotted with a primary antibody against SUMO1 (7341- Merck KGaA diluted 1:1000) followed by an appropriate secondary antibody (1:5000), prior to visualization by enhanced chemiluminescence (ECL, GE Healthcare, Munich, Germany). Quantitative densitometric analyses were performed using the Odissey acquisition system (LIC-OR Biosciences, Lincoln, NE, USA) and Geldoc Imaging (Bio-Rad, Hercules, CA, USA).

### 4.6. Protein Evaluation by SILAC and Mass Spectrometry

SMCs were grown for 15 passages in 35 mm Petri dishes in the presence of labeled (heavy)-Arginine (13C6 15N4), Lysine (13C6 15N2), or natural (light) amino acids. The samples were utilized after 95% enrichment was reached, as verified by mass spectrometry analysis. After a 20 h incubation with FCS 10% plus vehicle (DMSO, control cells) or with compound **12**, the cells were collected and lysed with M-PER (Mammalian Protein Extraction Reagent) buffer (Thermo Fisher Scientific, Waltham, MA, USA) plus protease and phosphatase inhibitors. Two biological replicates were analyzed: the first one in the so-called direct experiment; the second one in the reverse experiment. In detail, in the direct experiment, a lysate 1:1 mixture (Figure S3) of light-labeled control (L) and heavy-labeled furoxan-treated sample (H) was prepared. In the “reverse experiment”, the labeling was inverted. After protein determination, 10 ug of each sample was digested by the “Filter Aided Sample Preparation (FASP)” as suggested in [191]. The two lysate 1:1 mixtures (direct and reverse) were treated with dithiotreitol, iodoacetamide, trypsin, and, after desalting, injected in a nano-UPLC (Easy-nLC 1000, Proxeon Biosystem). Peptide separation was achieved using a reverse-phase silica capillary column (12 cm, packed with 1.9 µm ReproSil Pur 120 C18-AQ; flow 300 nL/min), with elution gradient A (0.1% *v*/*v* formic acid in water) and eluent B (0.1% *v*/*v* formic acid in acetonitrile from 0 to 45% B in 45 min, from 45 to 90% B in 2 min, and isocratic flow at 90% for 13 min). Mass spectrometry analysis was performed in positive polarity on a Q-Exactive mass spectrometer (ThermoScientific) equipped with a nanoelectrospray ion source (Proxeon Biosystems, Odense, Denmark). Full scan mass spectra were acquired with the lock-mass option, resolution set to 35,000, with 3 × 10^6^ as AGC value. The acquisition mass range for each sample was from m/z 300 to 2000. Tandem mass spectrometry analysis was carried out in a data-dependent acquisition mode using an HCD fragmentation. The ten most intense doubly and triply charged ions were selected and fragmented using a normalized collision energy of 27, a resolution set to 17,500, and an AGC equal to 1 × 10^5^. Target ions already selected for the MS/MS were dynamically excluded for 15 s. Every sample was analyzed in technical triplicates. MaxQuant software version 1.3.0.5 [192] was used for SILAC quantitation analysis with the following settings: briefly, searches were made against the UniProt_CP_human_proteome_ 20220525 (101,676 sequences; 41,413,969 residues) with 7 ppm as peptide tolerance, 0.5 Da for MS/MS tolerance, methionine oxidation and acetyl (protein N-term) as variable modifications, cysteine carbamidomethylation as fixed modification, two missed cleavages, Trypsin/P as cleaving agent, 1% FDR, and minimum peptide length of six amino acids for identification. Lists of 838 identified and quantified proteins were subjected to Perseus software analysis 1.5.5.3 [193] in order to define the proteins that were significantly up- and downregulated in a concordant manner in the two biological replicates (H/L ratios with significance B ≤ 0.05, upon Benjamini–Hochberg FDR correction). After an assessment of the quality of the experiment by normalization of the H/L ratios and by dot-blot representation using Perseus software, we also demonstrated that the labeling did not introduce perturbations to the system, since the data are extremely close to the 0 value [194]. The mass spectrometry proteomics data were deposited to the ProteomeXchange Consortium via the PRIDE [1] partner repository with the dataset identifier PXD043814 and 10.6019/PXD043814 [195].

### 4.7. Proteomics

Significantly differing MS-identified proteins were functionally correlated by the network building tool from the MetaCore (v. 22.4) integrated software suite for functional analysis (Clarivate Analytics, London, UK). MetaCore processing is based on a manually curated database of human protein–protein and protein–DNA interactions, signaling, and metabolic pathways in physiological and pathological conditions, from scientific literature and related databases. We previously proved the reliability of the MetaCore-obtained results by applying it to predict biomarkers and/or related pathways for defining the biochemical bases of the systems we investigated [196,197,198,199].

The gene names of the identified proteins were imported into MetaCore and processed using the “shortest path” algorithm. This builds hypothetical networks by cross-linking experimental factors that directly interact or that need a further factor, not present in the processed experimental list, but supported by MetaCore database, to be functionally correlated. Nets were built limiting protein process to individual proteins and excluding their involvement in multimeric complexes. The maximum number of allowed steps, cross-linking two experimental proteins via a software-added protein, was set to two and canonical pathways were avoided. The generated pathway maps were then prioritized according to their statistical significance (*p* < 0.001) and the most significant direct interaction and shortest path networks were graphically visualized as nodes (proteins) and edges (the relationship between the proteins). A wide search was then performed in PubMed/PubMed Central and Google Scholar for the functional review of the outlined protein cross-talks and pathways to define their possible role and outcome.

### 4.8. Data Analysis

Cell proliferation data were expressed as % of cell growth vs. control ± standard deviation (SD), calculated as a mean of the values obtained by three different Petri dishes, each measured in triplicate. Data were considered significant when the value of the Student’s t test for uncoupled samples was <0.05. The same analysis was applied for the densitometric analysis of the Western blots. In the SILAC experiments, the statistics on H/L ratios were run using Benjamini–Hochberg FDR correction. B values <0.05 were considered statistically significant. MetaCore statistics have already been illustrated in the appropriate section.

## 5. Conclusions

We found that, differently from classical NO donors, furoxans not only possess vasodilating properties after activation due to thiol-induced ring opening, but also inhibit SMC proliferation (dual effects). The range and tunability of their potencies are dependent on the electron-withdrawing nature of the substituent in position 3 of the ring. While vasodilation is NO-mediated, the inhibition of SMC proliferation, as documented from our proteomics studies, seems to depend on altered expression of several cell proteins. Among these, SUMO1, a control hub of cell homeostasis and whose activation plays a pivotal role in atherosclerosis by increasing SMC proliferation and promoting the switch towards a macrophage-like phenotype, is a target of furoxans. In the future, the pharmacological need may determine furoxan utilization: potent ones may be used as chemotherapeutics (antielmintics, antitumoral drugs), while those with a slower and more controlled ring opening may play a relevant role in atherosclerosis, a chronic pathology in which beneficial results may be achieved without the well-known risk of hypotension after the administration of classical NO donors. Animal models may help to elucidate these properties. Concerning the “culprits” of furoxans’ effect on SMC proliferation, we plan to identify the main intermediates generated by their degradation and to test them in vitro.

## Figures and Tables

**Table 1:** Effect of mono- and dinitrates on rat aorta strips vasodilation and human SMC proliferation.

Product Number	Structure	Vasodilating Activity EC_50_ ± SE (µM)	Antiproliferative Activity IC_50_ (µM)
1	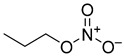	41 ± 6	n.c. ^1^
2	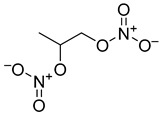	0.24 ± 0.03	>100
3	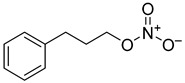	0.16 ± 0.03	n.c.
4	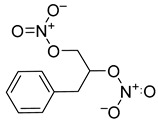	0.063 ± 0.010	n.c.

^1^ n.c., not calculated.

**Table 2:** Potency in rat aorta ring vasodilation and in inhibition of human SMC proliferation elicited by furoxans differently substituted in position 3 (R_1_) (products 5–14) and in position 4 (R_2_) (products 15–19).

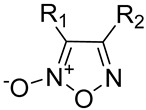				
Product Number	R_1_	R_2_	Vasodilating Activity EC_50_ ± SE (µM)	Antiproliferative Activity IC_50_ (µM)
5	PhSO_2_	OEt	0.012 ± 0.002	0.294
6	PhSO_2_	Ph	0.024 ± 0.003	1.89
7	CH_3_	Ph	146 ± 31	n.c.
8	Cl	Ph	0.98 ± 0.20	1.28
9	NO_2_	Ph	0.043 ± 0.006	2.82
10	NH_2_	Ph	14 ± 1	n.c.
11	COOCH_3_	Ph	0.16 ± 0.02	10.08
12	CN	Ph	0.0018 ± 0.0004	0.84
13	Ph	Ph	5.0 ± 0.7	47.36
14	CONH_2_	Ph	0.78 ± 0.08	14.83
15	Ph	PhSO_2_	0.053 ± 0.005	>100
16	Ph	CN	0.0043 ± 0.0005	>100
17	Ph	Cl	0.088 ± 0.011	>100
18	Ph	NO_2_	0.53 ± 0.09	Ineffective
19	Ph	COOCH_3_	1.4 ± 0.2	Ineffective

**Table 3:** Comparison between potencies on SMC proliferation elicited by 4-Ph ans and their corresponding 3-Ph furoxans and furazans.

4-Ph Furoxan Number and IC_50_ (µM)	3-Ph Furoxan Number and IC_50_ (µM)	Furazan Number and IC_50_ (µM)
5/0.294	-	20/uneffective
6/1.89	15/>100	21/uneffective
8/1.28	17/>100	22/uneffective
9/2.82	18/uneffective	23/uneffective
11/10.08	19/uneffective	24/uneffective
12/0.84	16/176.7	25/uneffective
13/47.36	-	26/uneffective
14/14.83	-	27/uneffective

## Data Availability

The mass spectrometry proteomics data have been deposited to the ProteomeXchange Consortium via the PRIDE partner repository with the dataset identifier PXD043814 and 10.6019/PXD043814.

## Supplementary Materials

The following supporting information can be downloaded at: https://www.mdpi.com/article/10.3390/molecules28155724/s1.

## Sample Availability

Not available.
