# Supplementary material for: Proteomics Studies Suggest That Nitric Oxide Donor Furoxans Inhibit In Vitro Vascular Smooth Muscle Cell Proliferation by Nitric Oxide-Independent Mechanisms
Source: Molecules. 2023 Jul 28;28(15):5724. doi: 10.3390/molecules28155724 (PMC10420201; doi:10.3390/molecules28155724)
Supplement: Supplementary file 1 [file molecules-28-05724-s001.zip › molecules-2451554-supplementary.pdf]

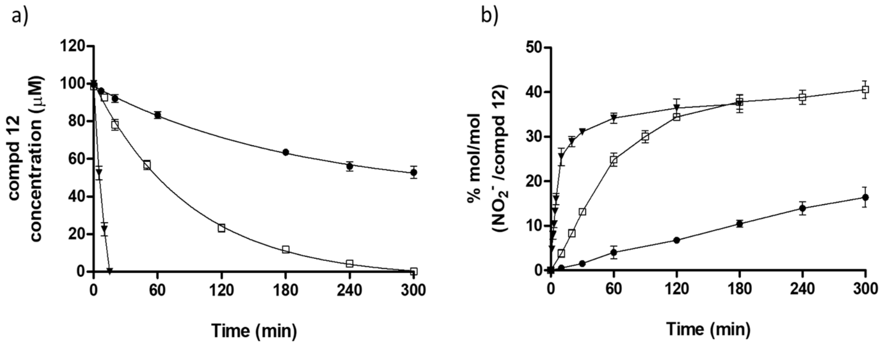

Figure S1. Reactivity and release of NO_2^- of 3-cyano-4-phenyl furoxan (compound **12**) in the presence of different concentrations of N-acetyl L-cysteine: 0.1 mM of compound **12** dissolved in PBS (0.05M, pH=7.4,1% DMSO) in the presence of N-acetyl L-cysteine 0.5mM (5x, ●), 5mM (50x, □), 0.1M (1000x, ▼) at 37°C during 5 hours. a) the results derive from HPLC analyses and are reported as compound **12** concentrations at various incubation times (mean ±SE); b) the results derive from Griess assay and are reported as % moles NO_2^- produced with respect to the moles of compound 12 incubated (% mol NO_2^-/mol compound) at various incubation times (mean ±SE).

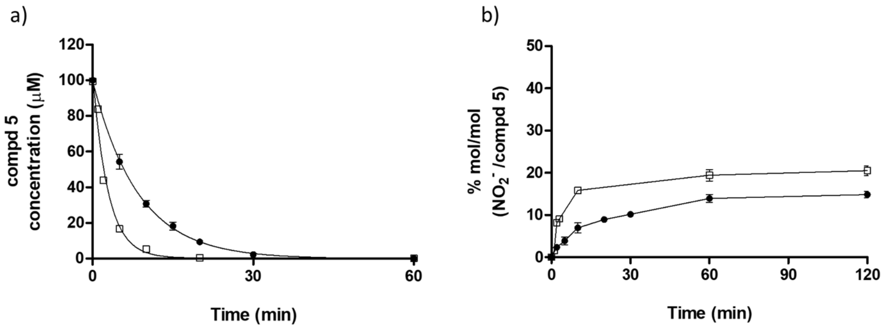

Figure S2. Reactivity and release of NO_2^- of phenylsulfonyl -4-ethoxy furoxan (compound **5**) in the presence of different concentrations of N-acetyl L-cysteine: 0.1 mM of compound **5** dissolved in PBS (0.05M, pH=7.4,1% DMSO) in the presence of N-acetyl L-cysteine 0.5mM (5x, ●), 5mM (50x, □), at 37°C during 2 hours. a) the results derive from HPLC analyses and are reported as compound **5** concentrations at various incubation times (mean ±SE); b) the results derive from Griess assay and are reported as % moles NO_2^- produced with respect to the moles of compound 5 incubated (% mol NO_2^-/mol compound) at various incubation times (mean ±SE).

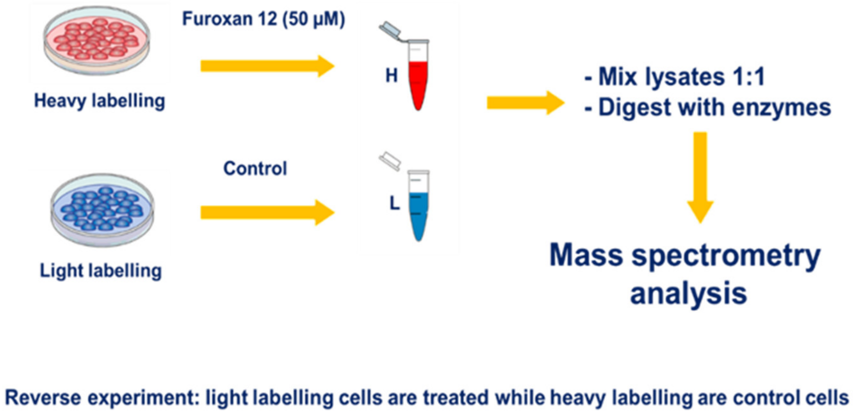

Figure S3. Protocol utilized for proteomics (SILAC): direct experiment.

Supplementary Table S1. Complete list of furoxans and related furazans tested for their ability to inhibit SMC proliferation. Experimental conditions as in figure 1.

Furoxans			Furazans
**R1**	**R2**	**IC_{50} (μM)**	**IC_{50} (μM)**
OC_2H_5	SO_2Ph	0.294	n.c.
Ph	SO_2Ph	1.89	n.c.
Ph	CH3	n.c.	n.a.
Ph	Cl	1.28	n.c.
Ph	NO_2	2.84	n.c.
Ph	NH_2	n.c.	n.a.
Ph	OCH_3	27.30	n.a.
Ph	$COCH_3$	7.52	n.a.
Ph	$COOCH_3$	10.08	n.c.
Ph	CN	0.84	n.c.
Ph	Ph	47.36	n.c.
Ph	$CONH_2$	14.83	n.c.
Ph	SCH_2CH_2OH	>100	n.a.
SO_2Ph	Ph	>100	n.a.
Cl	Ph	>100	n.a.
$COOCH_3$	Ph	n.c.	n.a.
NO_2	Ph	n.c.	n.a.
CN	Ph	176.7	n.a.

n.c. not calculated
n.a. not assayed
